# *I Me Mine*: on a Confusion Concerning the Subjective Character of Experience

**DOI:** 10.1007/s13164-016-0313-4

**Published:** 2016-05-27

**Authors:** Marie Guillot

**Affiliations:** Department of Philosophy, University College London, Gower Street, London, WC1E 6BT UK

## Abstract

In recent debates on phenomenal consciousness, a distinction is sometimes made, after Levine (2001) and Kriegel (2009), between the “qualitative character” of an experience, i.e. the specific way it feels to the subject (e.g. blueish or sweetish or pleasant), and its “subjective character”, i.e. the fact that there is anything at all that it feels like to her. I argue that much discussion of subjective character is affected by a conflation between three different notions. I start by disentangling the three notions in question, under the labels of “for-me-ness”, “me-ness” and “mineness”. Next, I argue that these notions are not equivalent; in particular, there is no conceptual implication from for-me-ness to me-ness or mineness. Empirical considerations based on clinical cases additionally suggest that the three notions may also correspond to different properties (although the claim of conceptual non-equivalence does not depend on this further point). The aim is clarificatory, cautionary but also critical: I examine four existing arguments from subjective character that are fuelled by an undifferentiated use of the three notions, and find them to be flawed for this reason.

## Introduction

### The Subjective Character of Experience

Experiences have intentional properties: they represent the world as being a certain way. Unlike other types of representations, they also have phenomenal properties: there is, as Nagel (1974) famously puts it, “something it is like” to have an experience for the subject undergoing it. Take the sentence “honeysuckle has a sweet smell”, and a sensory experience of the sweet smell of honeysuckle. There is an aspect of reality that both representations are *about*; yet they differ in that the existence of the sentence, in itself, doesn’t *affect* anyone in any particular way, while the existence of the experience affects me, the subject in which it occurs, by colouring my state of consciousness in a distinctive way – making it a flowery, fragrant, pleasant state to be in.

In recent debates on consciousness, a further distinction is sometimes made, following Levine (2001) and Kriegel (2009),^1^ between two dimensions of phenomenality itself. Levine calls them “qualitative character” and “subjectivity”, respectively; Kriegel uses the terms “qualitative character” and “subjective character”, a pair I will prefer for its uniformity. Here is how Levine introduces the two notions:

Let’s take my current visual experience as I gaze upon my red diskette case, lying by my side on the computer table. I am having an experience with a complex qualitative character, one component of which is the color I perceive. Let’s dub this aspect of my experience its “reddish” character. There are two important dimensions to my having this reddish experience. First, as mentioned above, there is something it’s like for me to have this experience. […] [B]eing an experience, its being reddish is “for me,” a way it’s like *for me*, in a way that being red is like nothing for—in fact is not in any way “for”—my diskette case. Let’s call this the *subjectivity* of conscious experience. […]

The second important dimension of experience that requires explanation is qualitative character itself. Subjectivity is the phenomenon of there being something it’s like *for me* to see the red diskette case. Qualitative character concerns the “what” it’s like for me: reddish or greenish, painful or pleasurable, and the like. (Levine 2001: 7.)

To notice the difference between these two dimensions is not to say that they are separable in reality; what is intended, rather, is a conceptual distinction between simultaneously present and interdependent facets of phenomenal consciousness. Within the complete phenomenal character of an experience – what it is like for me to have the experience – we can place the emphasis, and fix our attention, on two different aspects: the qualitative character – *what* it is like for me to have it – and the subjective character – what it is like *for me* to have it.

In Kriegel’s treatment, this comes out as a difference in levels of generality, or in how fine-grained we make our apprehension of phenomenality. At the most fine-grained end of the spectrum, I can focus on the exquisitely particular way it is like for me when I smell the fur of a wet dog, have pins and needles, or feel a pang of nostalgia. But I can also shift to the opposite end of the spectrum, noticing the general feature, across all of the above experiences, that *there is something or other that it feels like to me*. Whenever I am in a phenomenally conscious state, there is a subjective character to the experience; but depending on what particular experience it is, the subjective character will be specified *as* this or that qualitative character. As Kriegel puts it, subjective character captures what remains *constant* across experiences, while qualitative character captures what *changes*:

On the scheme I have adopted, bluish-for-me-ness, reddish-for-me-ness, trumpet-ish-for-me-ness, and so on are all phenomenal characters that are determinates of the determinable something-for-me-ness (or plain for-me-ness for short). One can focus the mind purely on subjective character by considering that which remains invariant among all the different determinates, and on qualitative character by considering that which varies among them. (Kriegel 2009: 54.)

Kriegel thus casts the difference between subjective character and qualitative character as a difference between a *determinable* and its *determinates*.

### Subjective Character by Other Names

In the literature on phenomenal consciousness, a wide range of alternative terms are used to talk about subjective character. We have already encountered, in the quotes of the previous section, *subjectivity* (Levine 2001) and *for-me-ness* (Kriegel 2009); others talk of *me-ishness* (Block 2007), *me-ness* (Block 1995), *myness* (Zahavi and Parnas 1998; Young 2008), or *mineness* (Zahavi 1999; Frith 1992); of a *first-personal character* or *first-personal givenness* of experience (Zahavi and Parnas 1998); of *non-reflective* or *pre-reflective self-awareness* (Zahavi 1999, 2005; Goldman 1970) or *low-level self-consciousness* (Flanagan 1992); of *the sense of self* (G. Strawson 1997; Damasio 1999) or *sense of ownership* (Zahavi, Gallagher 2004; Block 2007) attaching to experiences; and the list goes on. These terms tend to be treated as synonymous. Here is for instance Kriegel (2006: 205):

Assuming (plausibly) that Block’s notion of *me-ishness* is more or less the same as my notion of *subjective character*, or *for-me-ness* […]. (My emphasis.)

And here is the kind of passage from Block that might be taken to support Kriegel’s assumption of synonymy:

We may suppose that it is platitudinous that when one has a phenomenally conscious experience, one is in some way aware of having it. […] Sometimes people say Awareness is a matter of having a state whose content is in some sense *‘presented’ to the self* or having a state that is ‘*for me*’ or that comes with *a sense of ownership* or that has ‘*me-ishness*’ […]. (Block 2007: 484; my emphasis.)

In the same spirit, Zahavi and Kriegel (2015: 38) write:

[…] the point is that each of these objects [of experience], when experienced, is *given to one in a distinctly first-personal way*. […] To deny that such a feature is present in our experiential life, to deny the *for-me-ness* or *mineness* of experience, is to fail to recognize the very *subjectivity* of experience. (My emphasis.)

The following characteristic passage from Gallagher and Zahavi (2005/2014) uses no less than seven different expressions, treated as broadly equivalent, to capture the subjective character of experience:

The notion of **pre-reflective self-awareness** is related to the idea that experiences have a **subjective ‘feel’** to them, a certain (phenomenal) quality of ‘what it is like’ or what it ‘feels’ like to have them. […] [A]s I live through these differences [between various experiences], there is something experiential that is, in some sense, the same, namely, their distinct **first-personal character**. All the experiences are characterized by a quality of ***mineness*** or ***for-me-ness***, the fact that it is *I* who am having these experiences. […]. All of this suggests […] that (phenomenal) consciousness consequently entails **a (minimal) form of self-consciousness**. In short, unless a mental process is **pre-reflectively self-conscious** there will be nothing it is like to undergo the process, and it therefore cannot be a phenomenally conscious process […]. (My bold type; authors’ italics.)

However, this terminological luxuriance, I will argue, does not help to delineate the notion at stake precisely. On the contrary, it betrays a certain conceptual blurriness in the discussion. Despite the widespread assumption of a conceptual convergence, the same terms are being used by different authors (and sometimes by the same author in different places) to stand for very different interpretations of the notion of subjective character. Furthermore, this ambiguity amounts to a confusion, which is damaging to a number of current argumentative strategies.

### Goals

My goal in what follows is threefold: clarificatory, cautionary, and critical. Section 2, 3 and 4 are devoted to the clarificatory and cautionary parts. I start by showing in Section 2 that ‘subjective character’, and the family of terms widely treated as its synonyms, are used to cover at least three different notions, corresponding to three different ways to think of how the subject features in experience. For convenience, I’ll reserve for each of the three notions one of the expressions currently used indifferently: ‘for-me-ness’, ‘me-ness’, and ‘mineness’.

In Section 3, I go on to show that the three notions do not form an equivalence class: they do not stand in relations of mutual *a priori* entailment. In particular, for-me-ness entails neither me-ness nor mineness.

Section 4 is devoted to an empirically-informed discussion of pathological cases which might be taken to indicate that the corresponding properties do not always occur together. This would suggest that different *properties*, and not just distinct *concepts*, are being conflated in the debate. If this is correct, then my ternary conceptual distinction may open the way for a more accurate description of some pathological forms of experience than coarser-grained frameworks allow. This descriptive gain would provide further support for the threefold distinction.

Section 5 turns to the critical part. The conflation is problematic not just in principle, but because it makes a difference to existing arguments in the literature. I review four such arguments, and argue that they should be rejected.

## Three Notions

### Subjective Character and the Subject: From For-me-ness to Me-ness and Mineness

As this section aims to show, actual uses of the expressions listed above – ‘for-me-ness’, ‘me-ishness’, ‘mineness’, ‘first-personal givenness’, etc. – reflect an uncertainty as to what exactly remains constant across experiences.

One thing seems uncontroversial. The array of terms listed in Section 1 points repeatedly to the subject: “subjective”, “first person”, “self”, “me”, “my”, “mine”, and so on. The suggestion is that the subjective character of experience essentially involves the subject of experience.

This presumably has to do with the fact that subjective character, as the generic form of phenomenal character, is what characterizes experiences as such (by contrast with other types of representations); and that the existence of experiences in the world is inseparable from the existence of subjects in it. The defining feature of subjects, it is often assumed, is that they are not simply present in the world; the world is *presented to* them. Subjects are *parts* of the world, just like mountains, hurricanes and chairs, but the world also *appears to* them, or is ‘given’ to them, as it does not appear – is not ‘given’ – to mountains or chairs. But to “be appeared to’, for the subject, just is to have experiences, and for those experiences to have a subjective character. The point is often put by saying that a subject’s experiences constitute her ‘point of view’ on the world, and that having such a point of view is what it is to be a subject.

In short, subjects, experiences, and subjective character, can only be defined in terms of one another. However, this doesn’t tell us what exact form the involvement of the subject should take in the definition of subjective character.

Clearly, ‘subjective character’ is not merely a label for the *metaphysical fact* that experiences are had by subjects, in the sense that they occur *in* them. It is intended to capture a further, *epistemic fact*, namely that subjects are aware *of* their experiences; and, more demandingly still, a *phenomenological fact*, namely that this awareness is experiential, and registers as a certain “way it feels” to the subject.

This phenomenological thesis, however, still leaves open a number of ways in which the subject could feature in a description of subjective character. Under the pen of different writers, the subject appears variously:

- as one of the two *relata* in the relation of phenomenal awareness to her experiences, i.e. as the one who is *appeared to*;
- as also *appearing* to herself in being aware of the other *relatum* (the experience);
- as appearing to herself *as the owner* of the experience.

I briefly present the three options below.

### For-me-ness

To fix ideas, I’ll reserve the expression “for-me-ness” to label the first way in which subjective character is commonly talked about. On this first construal, the object of awareness is the experience itself.

#### A Special Awareness of Experiences

The notion can be introduced by observing that an experience has a manner of being unlike that of other types of things. This is partly because there is someone – a subject – to whom the experience is present in a special way, for whom there is something it is like for that experience to exist at all. And no one else is affected in the same way by the existence of that particular experience. The experience is in this sense *given to* someone; it is *for* that someone, to the exclusion of anyone else. Which amounts to saying that there is someone for whom the experience, by virtue of its mere existence and so long as it exists, is an object of a special sort of awareness:

a mental state has subjective character just in case it is for the subject, in the sense that the subject has a certain awareness of *it*. (Kriegel 2009: 38, my emphasis)

If I am feeling hungry or elated, you, too, can be aware of my hunger or elation, but not merely *by virtue* of the experience’s existence. Moreover, my own awareness of my experience is a way the experience *affects* me; it is a *phenomenal* kind of awareness. Not so with your awareness of my experience. You can’t grasp it “from the inside” (although you can of course have a – distinct – experience of the same type).

The familiar observations above suggest that, on this first interpretation, subjective character is simply another name for phenomenal consciousness, considered generically and under the most neutral description. As Kriegel puts it: “a conscious experience’s qualitative character makes it the conscious experience it is [e.g. a blueish or sweetish experience], while *its subjective character makes it a conscious experience at all*.”^2^

#### The “State-Self-Awareness” View

In this first reading of subjective character, what is at issue is the *subject*’s special awareness of her experience. However, according to several authors, the most plausible way in which this could obtain is through that *experience*’s awareness *of itself*. Whenever we are aware of something (a wet dog, a bird’s song, a flower’s fragrance), this is because a mental state of ours has that thing as its object. So, the reasoning goes, in the case where what we are aware of is our mental state itself, the same must be true, and the mental state must have *itself* as its object.^3^ The fact that a mental state is ‘for-me’ would thus be understood in terms of the state having an “inner awareness”, turned towards itself, in addition to constituting the subject’s awareness of something else, i.e. that which the experience is an experience of (the dog, the song, the fragrance, etc.).

Let us call the “*state-self-awareness view*” the view that subjective character, construed as the property which experiences have of being “for me”, is constituted by an awareness those experiences have of themselves.^4^ This thesis has different variants. The relevant kind of awareness is variously considered as a form of *representation* (Kriegel 2009); as a form of *acquaintance* (Williford 2015); or as a more elusive, *sui generis* “*intrinsic glow*”^5^ that makes the state aware of itself without the need for it to dissociate itself in an act-object structure (Husserl 1928/1964, Zahavi 1999; Drummond 2006). These differences, however, are immaterial to what follows. I will remain entirely neutral as to whether the state-self-awareness view in general, or in any of its variants, is true. I introduce the view here because it will play a role in the discussion in Section 3.5. What will matter then is the general claim that a state is “for-me” in so far as it is aware of itself.

To summarise this sub-section, subjective character construed as ‘for-me-ness’ is the special awareness a subject, and she alone, has of her own experience just by virtue of having it. State-self-awareness theorists believe this is the result of the experience being aware of itself. However this might be, though, what matters is that on the “for-me-ness” interpretation of subjective character, the object-side of the relation of awareness is taken up *by the experience*. The subject, for her part, features only on the “recipient” side of the relation of givenness that gives her access to her experience. Her place is reversed in the second construal, where she (also) features as the object of awareness.

### Me-ness

A second construal of subjective character places the focus of awareness on the subject herself, rather than (just) her experience. According to a widespread view, what makes an experience special for its subject is the fact that, in living through it, the subject is somehow aware *of herself*. Enjoying phenomenal consciousness is a way to be phenomenally *self*-conscious. I’ll reserve the expression “me-ness” as a label for this second interpretation.

The more general idea that consciousness involves self-consciousness on the part of the subject has a long history. Descartes, whose term for a conscious mental state is “thought”,^6^ argues in the *Second Meditation* that any thought necessarily entails a knowledge of myself. What is more, as he insists in the discussion of the *Seventh Objections* to his *Meditations*, this self-knowledge resides *in* the original conscious state:

[T]he initial thought by means of which we become aware of something does not differ from the second thought by means of which we become aware that we were aware of it, any more than this second thought differs from the third thought by means of which we become aware that we were aware that we were aware. (1996, vol. VII, p. 559)

Thus, it is at least arguable that for Descartes self-consciousness is a *constitutive* aspect of consciousness. This also may have been the view of Locke, according to whom it is “impossible for anyone to perceive, without perceiving, that *he* does perceive” (*Essay*, 2.27.9; my emphasis).

The idea of a constitutive link between consciousness and self-consciousness lives on in the works of Kant, Fichte, and Husserl. It is worth noting that neither Kant nor Fichte primarily envisaged a specifically *phenomenal* form of self-consciousness. The chief form of self-consciousness of interest to Kant, what he calls “transcendental apperception”, resides in a formal feature, “the form of the ‘I think’, that structures all experience without being an object of experience. Fichte for his part talks of an “intellectual intuition” of the “I”. Husserl, on the other hand, is a closer forebear to the contemporary notion of subjective character interpreted as “me-ness”. For Husserl, the “transcendental self” is directly given in experience: “I exist for myself and am constantly given to myself, by experiential evidence, as ‘I myself.’ […] [I]t is true, moreover, with respect to any sense of the word ego.” (1931/1960, Sec. 33).

This prefigures^7^ the claim, frequently found in the contemporary literature on subjective character, that when phenomenally conscious of anything, the subject is also phenomenally conscious of herself. In Zahavi’s words:

[…] phenomenal consciousness must be interpreted precisely as entailing a minimal or thin form of self-awareness. (Zahavi 2005: 16)

[…] if a certain organism is in possession of phenomenal consciousness, then it must also be in possession of both a primitive form of self-consciousness and a core self. (*Ibid*., 235–6).

Similar claims are found in Frankfurt (1988: 162); Flanagan (1992), Block (1995, 2007); Chalmers (1996); Siewert (1998); Burge (1998: 248), among many others.

The need for a distinction between for-me-ness and a second dimension of subjective character has been noted before. Neisser (2014), for instance, distinguishes “inner awareness” from “subjectivity”, arguing that “subjectivity is not best understood as inner awareness”.^8^ Drummond (2006: 199–200) and Sebastian (2012) make related distinctions. The binary distinction made in these works (and others) is a valuable one. However, it often consists in separating something like “for-me-ness” from a more “personal” dimension of subjective character which, I believe, still stands in need of further analysis. As I argue below, a finer, ternary distinction is required to pinpoint the confusion pervading the current debate, and to make an accurate description of a range of clinical cases.

### Mineness

A third common way of glossing subjective character presents it as a phenomenal awareness that my experiences are *mine*. I’ll reserve the term “mineness” for this third interpretation.

Everyone agrees that subjective character has to do with the fact that the existence of an experience resonates in a particular way with the subject in whom it occurs. While, on the first prominent way of discussing subjective character (as for-me-ness), it corresponds to the fact that the subject is aware *of her experience* (in a special way), on the third interpretation, it corresponds to the subject’s awareness of that very *fact*. As part of the experience being given to a subject, this subject is somehow aware of the experience *being her own*; i.e., of its being an object, for her, of that special sort of awareness we sometimes call “ownership”, or, to use the proprietary term, “for-me-ness”.

This interpretation of subjective character is more complex than the other two considered so far. It involves, in the subject of experience, (i) an awareness of the experience (as with for-me-ness); (ii) an awareness of herself (as with me-ness); and (iii) an awareness of the *relation* (of ownership, or for-me-ness) between the two.

The belief that the way an experience is “given” or “presented” to the subject marks, phenomenally, the experience as *hers* goes back at least to William James:

[T]houghts […] do not fly about loose, but *seem* each to belong to someone thinker and not to another. Each thought, out of a multitude of other thoughts of which it may think, is able to distinguish those which belong to its own Ego from those which do not. (James 1890, Chap. 10, 331 sq. My emphasis.)

In the recent debate, a similar idea is endorsed by Zahavi^9^:

One commonality [between different experiences] is the quality of mineness, the fact that experiences are characterized by first-personal givenness. That is, the experience is given (at least tacitly) as *my* experience, as an experience I am undergoing or living through […]. (Zahavi 2005: 16. My emphasis.)

Block (1995, 4.2.1) argues that animals lacking a concept of self, like dogs, still have a “self-orientation”, because their experiences are phenomenally presented as *theirs*:

Even if monkeys and dogs have no [conceptualised] self-consciousness, however, no one should deny that they have P[henomenally] conscious pains […]. P[henomenally] conscious states often seem to have a “me-ishness” about them, the phenomenal state often represents the state as “*a state of me*”. (Block 1995: 235. My emphasis.)

To sum up: construed as “for-me-ness”, subjective character is a subject’s characteristic awareness *of her experience*; read as “me-ness”, it is her awareness *of herself,* gained as part of having the experience; understood as “mineness”, it is her awareness of herself *as* having the experience.

## That the Three Concepts are not Equivalent

There is a clear *prima facie* distinction between the three notions: it is intuitively different to talk of an awareness of an experience, of an awareness of the experiencer, or of an awareness of the experiencer as owner of the experience.

### Formal Differences

Notice, moreover, the contrast in the form^10^ of the three predicates. The common talk of ‘subjective character’ as a “quality” or “feature” our experiences *have* or *come with* suggests a monadic predicate, of the form F(*x*) (where ‘*x*’ ranges over experiences). Understood as for-me-ness, however, subjective character would seem instead to assume the form of a *relation*: a relation R_1_ of awareness between a subject *s* and an experience *x* of hers, of the form R_1_(*s,x*). Understood as me-ness, subjective character appears to correspond to a relation R_2_ of a different form, R_2_(*s,s*): namely, a reflexive relation of awareness a subject *s* has to herself (while having an experience). Lastly, understood as mineness, subjective character should correspond to a different relation again, R_3_. Here we should expect a relation of higher complexity than the previous two, since it is a relation of awareness between subject *s* and a *fact*, i.e. the fact that she owns the experience she is contemplating. This might be spelled out as R_3_(*s*,[R_1_(*s,x*)]), i.e. as a relation R_3_ of awareness between a subject and the fact of ownership (the fact that experience *x* is “for her”). Clearly, R_1_, R_2_ and R_3_ have very different structures, and this in itself should make us wary of collapsing the three notions into one.

What I want to argue now is that, in addition, the three notions are not conceptually equivalent, since they do not stand in relations of mutual implication.

### Mineness and its Implications

It might seem that mineness, at least, should entail both me-ness and for-me-ness, in virtue of its more complex structure. As noted above, to become phenomenally aware of myself as the owner of a given experience, I need the more basic awareness of myself and of the experience. This could be, however, a slightly misleading way to speak, and much depends on what we mean here by “awareness”, i.e. whether or not we give it the narrower sense of a *phenomenally* conscious awareness.

Take a different kind of complex phenomenal state, like the visual impression of a piano. To achieve the three-dimensional awareness of the piano, I certainly need more basic one-dimensional information about lines, two-dimensional information about surfaces, and “two-and-a-half dimensional” information (Marr 1982) corresponding to the projection of the volume on the flat surface of the retina. But does this entail that, in being phenomenally aware of the piano as having depth and volume, I am also *phenomenally* aware of it as a two-dimensional, let alone a 2.5-dimensional object? That the one-, two- and 2.5-dimensional information is necessary to construct the phenomenally conscious, visual awareness of the piano occupying space doesn’t mean that this information itself is presented in a phenomenal format, as the final output is. And it is indeed quite unclear whether one can shift the focus of one’s attention, within the total visual impression of the piano, between, say, a 3-D, a 2.5-D and a 2-D appearance, as one can shift attention between the whitish and the blackish components in one’s impression of the keyboard. That we can do the first kind of shift is doubtful, even though we *can* perfectly well, in other contexts, have a 2-D phenomenal awareness of a piano (e.g., by looking at a drawing of the instrument). The complexity of a phenomenal datum does not always entail the *phenomenal* accessibility of each of its components. Thus, even if *some form or other* of awareness of the experience, and of myself, is a necessary condition for the *phenomenal* awareness of myself as owner of the experience (mineness), it doesn’t follow that an *a priori* relation of implication holds from mineness to for-me-ness (a *phenomenal* awareness of the experience) and me-ness (a *phenomenal* awareness of the experiencer).

### Me-ness and its Implications

It is much more doubtful still, in any case, that any other relations of entailment unite the three notions. Let us consider me-ness. I described it above as the putative phenomenal awareness of myself that I gain in having an experience. Does this not involve, at the very least, an awareness of the experience itself, i.e. for-me-ness?

A certain kind of transparency argument might make room for questioning this transition. Some^11^ writers suggest that when I am asked what it is like, exactly, for me (to smell a wet dog, to hear the song of the swifts), I must attend, in answering, to the same outward objects and properties as I would attend to if I were asked a question about dogs, or birds. This would suggest that what is “given to me” or “for me” in an experience is not the experience itself, but the worldly objects and properties that it represents. Thus Martin (2002) on staring at a lavender bush:

When my attention is directed out at the world, the lavender bush and its features occupy centre stage. It is also notable that when my attention is turned inwards instead to my experience, the bush is not instead replaced by some other entity belonging to the inner realm of the mind […]. I attend to what it is like for me to inspect the lavender bush through perceptually attending to the bush itself while at the same time reflecting on what I am doing. So it does not seem to me as if there is any object apart from the bush for me to be attending to or reflecting on while doing this. (Martin 2002: 380–1.)

Such a “transparentist” view – considered here merely for the sake of argument –might be compatible with a conception of subjective character as me-ness in which the latter does not entail any direct phenomenal awareness of the experience itself. Take my total experience as I play the piano. I am aware of the piano in front of me, and of the unfolding tune; and, even though this is not the focus of the experience, I am also continually aware of myself (my fingers on the keys, my feet on the pedals, my breathing, the contraction of my muscles). The transparentist could accept that I am thus presented with myself throughout, that there is in this sense a “me-ness” to the experience; she could even entertain the possibility that this might be the case whenever I experience; but she would at the same time deny that there is also a way in which I am presented with the experience itself. This is not conceptually incoherent. Accordingly, it is at least open to question whether the notion of me-ness (simply construed as the phenomenal awareness of myself that I gain through an experience) entails the notion of for-me-ness.

It is even more doubtful whether me-ness entails mineness. This would require a phenomenal awareness, both of myself and of my experience, and we have just seen that the latter part might not be secured *a priori* by the notion of me-ness. But it would also require something further, namely a phenomenal awareness of the *relation* of ownership between my experience and myself. Even granting that experience provides phenomenal access to both the subject and the experience itself, there is no *a priori* reason why it should also provide phenomenal access to the fact that the latter stands in a special relation to the former. In fact, in some extreme situations, like the pathological phenomenon of “inserted thought” in shizophrenics, there might be reason to think that phenomenal awareness of just such a relation is missing – more about this in Section 4.

If it is at least open to question whether either me-ness, or mineness, entail any of the other two notions (insofar as hypotheses under which the entailments don’t hold are not obviously incoherent), then there is in any case no *straightforward* implication to rely on. The transition from me-ness or mineness to the other notions should thus not be made without argument, as it so often is.

### For-me-ness and its Implications

More decisive than those doubts concerning the implications of mineness and me-ness, however, is the lack of any reason to think that the notion of for-me-ness should entail either me-ness or mineness. (Absent, that is, very substantial theoretical assumptions; more on this in Section 3.5.)

For-me-ness is a certain kind of awareness relation, connecting the subject to her experience, and perhaps, more primitively, the experience to itself (if the state-self-awareness thesis is correct). What matters is that in either case, only the *relatum* that isn’t the subject of the awareness figures as the *object* of awareness. The notion of an awareness of an experience will not, as such, yield the notion of an awareness of the self. The experience and the self are distinct particulars.^12^ And it is not generally the case that, when we say that something is “for me”, is given to me, we mean that I am aware of that thing *and also* of myself. Take the conscious perception of a table. When we say that this is a way that the table is given to me, we mean to talk about an awareness that has the *table* as its object. Without further assumptions being built into the notion of for-me-ness, i.e. without going beyond the terms in which it is usually introduced – as an awareness of a certain kind *of our experiences* – we shouldn’t accept that what goes for tables doesn’t go for experiences. When a situation is described as one in which things are “for me”, what is asserted is that I’m aware *of those things* (and not, *eo ipso*, of myself). I am *in* an awareness relation to those things; but this is not the same as being aware *of* the relation itself, or of the fact that it obtains. This relation of awareness as such doesn’t make me aware of both *relata*, but only of the *relatum* that isn’t me.

In the “for-me-ness” construal of subjective character, then, the self, to borrow an evocative expression often used by Zahavi and Drummond, is a mere “dative” of the experience. Only in the “me-ness” and perhaps in the “mineness” readings is it (also) involved in the “accusative” position, as an object of phenomenal awareness. To move from the dative to the accusative is to shift to a different notion. Kenneth Williford makes the point eloquently^13^:

If we accept that there is a dative of manifestation, that objects and qualities appear *to* someone or something, we are closer to but not quite up to subjective character just yet. Subjective character, recall, is supposed to be something phenomenologically detectable. And one might raise the following sort of worry. Suppose phenomenally manifest objects and properties are manifest *to* something or someone. It does not follow from this alone that that *to which* they are manifest is itself manifest or even manifestable. Nor does it follow that the *fact that* they are manifest to something is manifest or even manifestable. In other words, there could indeed be a dative of manifestation and yet no direct phenomenological evidence of this at all. Williford (2015: 9/27)

Now, one might object to the foregoing by insisting that for something to be “given to me” or “for me” isn’t just for me to be enjoying just *any* kind of awareness of it. It’s a matter of being *phenomenally conscious* of that thing; of it being *presented* to me, *manifest* to me – in short, of my *experiencing* it. And one might think that *this* kind of awareness relation, unlike others, does involve being aware of both *relata*.

To which I would reply that, while this might well be true, it is a substantive claim, and something for which evidence should be produced, rather than an immediate conceptual truth flowing from the notion of for-me-ness, which one could take for granted. The notion of “for-me-ness” that we started with, the pre-theoretical notion that is introduced through the familiar point that a subject is aware of her present experience in a way that others are not, doesn’t by itself yield any suggestion that part of what makes this “way of being aware” special is that it encompasses all of the subject of awareness, the object of her awareness and their relation within its reach. Nor does it follow from the grammar of such expressions as something being “given to *x*” or “for *x*” that *x* should at the same time be given to herself. I also doubt that such implications could be drawn from a mere conceptual analysis of the notion of “experience”, defined as a state there is something it is like to be in for its subject.^14^ Unless some non-trivial theoretical preferences are added to the notion of for-me-ness, then, there is no justification for using it interchangeably with the notions of me-ness and mineness, as is too often done without argument.

### For-me-ness and its Implications on (Some) State-Self-Awareness Views

What kind of further theoretical commitments could conceivably license the transition from for-me-ness to me-ness and mineness?

#### Maximalism About For-me-ness

Perhaps an example could be found in a possible variant of the state-self-awareness view, sketched above in Section 2.2. According to the state-self-awareness view, the for-me-ness of an experience (that I am aware of it in a special way) is a result of its being, more primitively, aware of itself. This is the view that Kriegel (2009), Williford (2015) and Zahavi (2005) defend under various specifications, as we have seen. One *could* conceivably interpret the claim common to all those variants – that a state that is “for me” is a state that is aware of itself – in a maximalist way, as meaning that such a state is aware of *all* of its properties, including the relational property of being an experience of this or that subject. However, this would not be a particularly attractive theory to defend; phenomenal awareness is not, in general, an awareness of all the properties of its objects, including their relational properties. (The visual awareness of a chair does not include an awareness of its relational property of having been made by a particular carpenter, or of being comfortable for me.) To my knowledge, none of the advocates of the state-self-awareness view endorses this extreme version.^15^ In any case, were one to defend such a maximalist version of the state-self-awareness view, this would constitute a substantive step beyond the mere use of the notion of for-me-ness, and it would be very different from assuming a conceptual equivalence between the three notions of subjective character.

#### Minimalism About the Self

Another example of a non-trivial assumption under which for-me-ness might entail at least me-ness is to be found in the “minimalist” approach to the self.^16^ A family of writers, including Zahavi (2005) and Williford (2015), argue that the self, or at least *a* form of selfhood (the “minimal self” or “core self”), is identical either with experience, or with some part or intrinsic property of experience.^17^ Williford (2015: 2/27) thus proposes that “we identify the subject with the episode or stream of consciousness itself”. Zahavi claims that the subject or self is identical with a *feature* of experience, namely its “givenness”:

[…] the self is […] identified with the very first-personal *givenness* of the experiential phenomena. […][T]he most basic form of selfhood is the one constituted by the very self-manifestation of experience […]. Thus, the self referred to is not something standing beyond or opposed to the stream of experiences but is rather a feature or function of its givenness. (Zahavi 2005: 106)

Now, on such views, it could be argued that for-me-ness *does* entail me-ness: if the self *is* the experience (or a part or intrinsic feature thereof), then to be phenomenally aware of the experience (for-me-ness) is to be phenomenally aware of the self (me-ness). Indeed, this is just the way Williford (2015) seeks to bridge the gap between for-me-ness and me-ness he so lucidly identifies in the passage quoted above.^18^ ^19^

However, the entailment between for-me-ness and me-ness, in this context, does not flow from a conceptual equivalence between the two notions, but from the additional support of a substantive extra premise. Namely, that the self is identical with (all or part of) the experience. This is in no way included in the notions of for-me-ness and me-ness, which merely stand for special relations of awareness between the subject and her experience, and between the subject and herself, respectively. No further commitment about the metaphysics of subjects is built into those concepts. And the minimalist thesis about the self is very far from trivial. According to common sense, and in ordinary linguistic practice, I *have* experiences (“I had a really bad experience with her last night”); it can be the case that I *had* an experience an hour or a year ago, now gone (“Visiting Venice was a wonderful experience”). This is incompatible with my *being identical with* the experience. The minimalist view isn’t uncontroversial in the context of philosophical thinking either. It competes, at the very least, with views according to which the self is a mental substance, a substratum *of* experiences (Descartes); an animal with both physical and mental properties (Olson, Snowdon); a spatial part of an animal, typically a brain or part of a brain (Parfit); a causal system that can produce experiences (Dainton, Peacocke); and so on and so forth. Thus, while the minimalist view of the self is a framework in which for-me-ness arguably entails me-ness, the entailment isn’t a matter of conceptual equivalence between those two notions, but depends on substantive extra premises in need of independent argument.

This last point will have some importance for the critical discussion in Section 5 below. Again, even on a minimalist approach, the implication from for-me-ness to me-ness depends on the minimalist view of the self being independently proved true. To be allowed to draw the implication, we need a separate defence of the thin metaphysics of selfhood. Instead of which, minimalists typically *assume* that the implication from for-me-ness to me-ness holds, because they often use an undifferentiated notion of subjective character to cover both aspects,^20^ and then go on to rely on the alleged conceptual connection to defend a thin theory of the self. This is getting it backwards, and the move is unwarranted.^21^

### A Tentative Diagnosis for the Assumption of a Conceptual Equivalence

For-me-ness, me-ness and mineness, then, do not form an equivalence class of notions. Whether mineness entails either for-me-ness or me-ness, or whether me-ness entails either for-me-ness or mineness, is at the very least open to question. In any case, it is clear that for-me-ness entails neither me-ness nor mineness, which is enough to defeat the assumption of mutual equivalence.

#### The Connotations of “Self-Awareness”

A number of considerations might help explain why this should be sometimes overlooked. One possible reason could be the ambiguity of the word “self”, which, as a reflexive pronoun, can stand for any reflexive relation, but which, as a noun, stands for a certain kind of particular: a subject. “Self-awareness” can thus mean the awareness that some particular (say an experience) has of itself, or, more specifically, the awareness that a *self* has of herself. But slips might occur between the two notions, encouraging, particularly under the assumption that for-me-ness is a matter of state-self-awareness, an unwarranted transition from for-me-ness to me-ness. The passage from Gallagher and Zahavi (2005/2014) already quoted above could well be an illustration of this, or is, more likely, at least guilty of a form of expression that invites the confusion, as it goes in the same breath from a “self-consciousness” understood as a “non-observational access to *myself*”, to a “*mental state*” being “self-conscious”^22^:

All the experiences are characterized by a quality of *mineness* or *for-me-ness*, the fact that it is *I* who am having these experiences. All the experiences are given (at least tacitly) as *my* experiences, as experiences *I* am undergoing or living through. All of this suggests that first-person experience presents me with **an immediate and non-observational access to myself**, and that (phenomenal) consciousness consequently entails a (minimal) form of **self-consciousness**. In short, unless **a mental process is pre-reflectively self-conscious** there will be nothing it is like to undergo the process, and it therefore cannot be a phenomenally conscious process […]. (My bold type.)

#### The Connotations of “First-Personal Access”

Another likely influence is the frequent use in the debate of the notion of a “first-person” access to, or perspective upon, or knowledge of, our own experiences, to capture their for-me-ness. The “first-person” qualifier just means that the subject has the exclusive enjoyment of this type of access, perspective, or knowledge; not that she is their object. By contrast, a “third-person” access, perspective or kind of knowledge are ones that are equally available to all thinkers. The asymmetry between first-person and third-person epistemic relations isn’t defined by what occupies the object side of the relation, but by the constraints on those – just me, or anyone – who can be on the recipient side. And while the self is certainly a salient candidate for being a possible object of first-personal access, not all first-person access is directed to the self; occurrent experience constitutes another such object. Still, the ambiguity in the surface-grammar of the expression “first-person perspective” – a perspective *of* the first person, or *on* the first person? – encourages reading off the notion more than it contains.

An interesting passage from Kriegel (2004) could constitute an illustration of this. Kriegel (2004, Section 4) defends the claim that intransitive consciousness depends on intransitive self-consciousness (i.e., on a peripheral consciousness the subject has of herself). In effect, this amounts to the claim that there can be no for-me-ness (the type of awareness of experiences that makes them conscious at all) without me-ness. The first of his two arguments,^23^ says Kriegel,

[…] can be summarized as follows: conscious states are first-person knowable; first-person knowable mental states must be intransitively self-conscious; therefore, conscious states are intransitively self-conscious. (2004: 198)

The decisive premise is the second one: that first-person knowable mental states must be states in which the self is intransitively (i.e. peripherally) conscious of herself. And this is just where the connotations of the “first-person” qualifier might be playing a role. Here is the argument Kriegel gives to establish the second premise:

Now, it seems that the only experiences and thoughts we can have first-person knowledge of are experiences and thoughts we have self-consciously, that is, experiences and thoughts we are peripherally aware of *having*. For when we have a mental state un-self-consciously – *that is, without any awareness of it whatsoever* – we have to infer its existence on the basis of evidence, which means that our knowledge of it is mediated in a way first-person knowledge is not. (2004: 198. My emphasis.)

Here is why I think the argument is problematic. The first sentence is a reformulation of the second premise: *one can’t have first-person knowledge of a state without being aware of having it*. In other words: given a mental state of which I know, if I have first-person knowledge of it, then I am aware of the fact that *I* am having it. This is then defended in the second sentence *via* an appeal to the intuitiveness of its contraposition, i.e. the proposition that, given a mental state of which I know, if I don’t have this knowledge in a way that involves self-consciousness on my part, then I don’t have first-person knowledge of it (but only evidence-based, third-person knowledge). But notice the gloss of the antecedent of this contraposition, inserted between the dashes (and italicised by me) in the quote above: not having knowledge of a mental state in a way that involves self-consciousness is suddenly equated to being “without any awareness of it whatsoever”. This is clearly *not* an equivalence that can be taken for granted, as it constitutes precisely what is at issue here, i.e. whether I can be phenomenally aware of my mental state without also being aware of myself. Once we accept the equivalence, the argument goes through, because a state of which I am not phenomenally aware is indeed a state I can only know about third-personally; but we shouldn’t accept it, because it’s question-begging.

So why is the equivalence put forward? I think it might inherit undue intuitive appeal from the connotations of two of the expressions used in the argument. First, knowing the experience in a way that involves being self-conscious is rephrased in the first sentence of the passage as *being aware of having* it. The surface grammar of the latter form of words might invite an equivocation. The most natural, and correct, way to read the infinitive clause is with an implicit subject or “PRO” argument (Higginbotham 2003), i.e. as “being aware of *my* having it”, which does express self-consciousness. However, because the subject is elided, the turn of phrase surreptitiously makes available another, weaker reading, namely, “being aware of the state being had”, i.e. being aware of its occurring. That a state I am not aware is being had (or is occurring) is not a state I am (phenomenally) aware of is trivially true; but that a state I am not aware of in a way that involves self-consciousness is a state I am not aware is being had at all is, on the other hand, a substantive claim: precisely the claim that the argument seeks to establish, and precisely the claim, too, that the problematic equivalence between the dashes smuggles in, possibly under the influence of the ambiguity in the expression “being aware of having it”.

Second, as pointed out above, the expression “first-person knowledge”, while really standing for the kind of knowledge only the subject is in a position to gain, has the potential to mislead, suggesting simultaneously that the subject is what the knowledge is *of*. Under this – unwarranted – suggestion, the claim that a subject who isn’t self-conscious as part of having an experience is unable to know the experience first-personally gains an unearned appearance of intuitiveness.^24^

## Different Concepts, or Different Properties?

The upshot of the previous section is that there is no mutual equivalence between the three notions really being conflated in debates on subjective character. At best, mineness might entail both for-me-ness and me-ness, but not as straightforwardly as it might initially seem. For-me-ness and me-ness are likely to be mutually independent, and neither depends on mineness. For-me-ness certainly entails neither me-ness nor mineness.

This is reason enough to avoid using the three concepts interchangeably. But could it be that they also apply to distinct properties? One way to show this would be to find phenomena in the actual world to which not all three notions are applicable. Some empirical cases might tentatively be taken to suggest that for-me-ness, me-ness and mineness can fail to occur simultaneously in some pathological conditions. In particular, me-ness or mineness may sometimes fail to be instantiated even though for-me-ness is instantiated. If me-ness and mineness can indeed selectively disappear, giving rise to distinct pathologies, then my threefold distinction might prove a useful tool for describing empirical data more accurately.

### For-me-ness Without Me-ness and Mineness?

In this sub-section, I rely heavily on recent work by Alexandre Billon (Billon 2014, forthcoming), elaborating on it slightly.

The first type of empirical case of potential relevance is the depersonalisation syndrome (Simeon and Abugel 2006; Sierra 2009), as well as its extreme, delusional form, first described by Cotard (1880). Patients who experience depersonalisation, typically as a part of severe depression, report an alteration of their experiences. In many cases, it no longer seems as though the experiences are *theirs*. And often, it no longer seems as though *they* (the patients) exist at all.

Here are two typical reports of the first kind of distortion: some depersonalised patients appear to be phenomenally aware of their experiences, but not of the fact that the experiences are *theirs*:

It was as if it was not me walking, it was not me talking, as if it was not me living […] I can look at me, I am somehow bothered by my body, as if it wasn’t me, as if I lived on the side of my body, on the side of myself if you like. I don’t know how to explain. (Janet and Raymond 1898: 70)^25^

When a part of my body hurts, I feel so detached from the pain that it feels as if it were somebody else’s pain. (Sierra and Berrios 2000: 163)

Here are also reports of “feelings of non-existence”, as Billon calls them. Billon interprets those as experiences in which the depersonalised patient lacks a phenomenal awareness of *herself*:

I imagine myself seeing life as if it were played like a film in a cinema. But in that case where am I? Who is watching the film? (Simeon and Abugel 2006: 15)

It almost feels like I have died, but no one has thought to tell me. So, I’m left living in a shell that I don’t recognize any more. (Sierra 2009: 27)

In a recent series of articles, Billon makes a powerful case for the claim that the experiences of depersonalised and Cotard patients is evidence of the possibility of phenomenal awareness without subjective character. But this claim might strike some as counter-intuitive, insofar as subjective character is supposed to be what makes something an experience at all. While I find the general direction of Billon’s argument very compelling, I think that the three-way distinction I propose in lieu of the misleadingly monolithic notion of subjective character might allow for a more conservative, and perhaps more intuitive, description of the pathology.

For consider: the first type of report above suggests that the patients lack a phenomenal awareness that the experiences they report are theirs (“I feel so detached from the pain that it feels as if it were somebody else’s pain”). In my terminology, they lack “mineness”. The second type of report suggests that those patients additionally lack a phenomenal awareness of the presence of their own selves (“It almost feels like I have died”; “where am I?”). As Simeon and Abugel (2006) put it, in depersonalisation “[there is] no clear feeling of ‘I’ (p. 25)”; “There [is] literally no more experience of a ‘me’ at all (p. 143–4)”. In my terminology, the depersonalised patients lack “me-ness”. On the other hand, *something* of the subjective character of the abnormal experiences does seem to be retained. Those experiences are “for” the subjects undergoing them, given to them in a special way that enables them to report on a phenomenal occurrence (a pain, an impression as of a walking movement, etc.) in the direct manner that no one else could report it. In my terminology, those experiences have “for-me-ness”. I thus propose to describe the depersonalisation syndrome as a condition in which experience lacks both mineness and me-ness, but retains for-me-ness.

A word on the motivation for locating the manifestation of the disorder at the *phenomenal* level. As Billon stresses, while Cotard patients are delusional, actively denying ownership of their experiences and sometimes claiming to be dead, depersonalised patients refrain from taking the abnormal experiences at face-value. They don’t actually *believe* that they don’t own the experiences, or that they don’t exist, as evidenced by the careful wording of their reports (“it was as if”; “it feels as if”; “it almost feels like”; “I imagine myself seeing”; “somehow”; etc.). As Billon convincingly argues, this makes it plausible that the problem comes specifically from the disturbing lack of an *impression*, of an *experience* that used to be present – rather than, say, from the lack of a *belief* (that the self exists or that the problematic experiences are hers).

### For-me-ness and Me-ness Without Mineness?

The second type of case of potential interest is the phenomenon of “inserted” or “implanted” or “alien” thoughts in some schizophrenic patients (Jaspers 1963; Frith 1992; Gallagher 2004; Bortolotti 2010). Inserted thoughts are thoughts that the subject reports as occurring in her stream of consciousness, but which, nonetheless, she refuses to acknowledge *as her own*, typically claiming that someone else produced those thoughts and put them in her mind. Here are some characteristic patients’ reports:

[S]he said that sometimes it seemed to be her own thought ‘but I don’t get the feeling that it is’. She said her ‘own thoughts might say the same thing’, ‘but the feeling isn’t the same’, ‘the feeling is that it is somebody else’s.’ […] (Allison-Bolger 1999, #89)

I look out of the window and I think the garden looks nice and the grass looks cool, but the thoughts of Eamonn Andrews come into my mind. There are no other thoughts there, only his… He treats my mind like a screen and flashes his thoughts on to it like you flash a picture. (Mellor 1970: 17)

One evening one thought was given to me electrically that I should murder Lissi. (Jaspers 1963: 580)

It is a much-debated question how thought insertion is best described and understood. What I will call the ‘simple account’ interprets it as a case where a subject lacks a “sense of ownership” over her thoughts (Metzinger 2003: 445–6). What is now the standard account treats it, rather, as a case where she *does* have a “sense of ownership”, but no “sense of agency”, over her thoughts, i.e. where she lacks the sense of having actively produced them (Stephens and Graham 1994; Gallagher 2004). A third account construes alien thoughts as cases where the subject fails to endorse the content of her thoughts or commit to them (Bortolotti 2010).^26^

A reason which is often given to reject the ‘simple account’ – which construes inserted thoughts as mental states unaccompanied by a feeling of ownership – is that the patient retains *first-personal access* to her inserted thoughts, just as to all of her other, normal thoughts. The “alien” thoughts do occur in her stream of consciousness, as she wouldn’t deny.

This case for rejecting the ‘simple account’, I suspect, rests in part on a failure to distinguish between different notions of subjective character. As I pointed out at the start, the concept of a “sense of ownership” is generally used in a fashion that doesn’t discriminate between: (i) a special way we are aware of our experiences – *first-personally*, i.e. in a way others can’t be aware of them; (ii) a way we are aware of *ourselves* in having experiences; and (iii) a way we are aware of *owning* those experiences. Once those three sense of “subjective character” are disentangled, more fine-grained conceptual resources become available to describe the predicament of a patient with alien thoughts. In particular, it becomes possible to acknowledge that the abnormal thoughts are given to her in a first-personal way (thus exhibiting *for-me-ness*), while still hypothesising that she might be specifically lacking a sense of *owning* those thoughts (*mineness*). Note that schizophrenics with thought insertion nonetheless seem to retain a phenomenal awareness of *themselves* (*me-ness*) while having the problematic thoughts, as shown by the normal use of first-person terms in their reports.^27^

In what amounts to a more sophisticated version of the ‘simple account’,^28^ made available by my threefold distinction, I thus propose to describe thought insertion as a condition in which experience lacks mineness, but retains both for-me-ness and me-ness.

### The Descriptive Benefit of the Tripartite Framework

My three-way distinction has the additional merit of providing us with a principled way to distinguish between the two types of pathologies discussed in the last two sub-sections, i.e. the depersonalisation spectrum and the phenomenon of inserted thoughts.

It is now customary to describe delusions as broadly rational responses to abnormal experiences (Davies and Coltheart 2000). *Which* abnormal experience helps us classify the delusions. In particular, the thought-insertion delusion and the depersonalisation syndrome in its delusional form (Cotard syndrome) can both be viewed as rational responses to unusual experiences, whose abnormality specifically has to do with an impaired subjective character.

But saying only this much leaves us without a way to mark the difference between the two conditions. Further distinguishing, under the blanket expression “subjective character”, the three notions I separate here, permits to characterise the two pathologies in distinct ways. I proposed that the experience of prototypical Cotard patients exhibits for-me-ness, *but neither me-ness nor mineness*. I also suggested that schizophrenic inserted thoughts might exhibit for-me-ness and me-ness, *but not mineness*. Why think that me-ness is lacking in the first type of case, but not the second? First, we should by default accept at face-value the reports of patients, and we have seen that “feelings of non-existence” are a specific complaint of Cotard sufferers. Here is a characteristic report by a patient: “I am not Myself at all. What is missing is myself, it is awful to elude oneself, to live and not to be oneself.^29^” Schizophrenics with thought-insertion do not make such reports of “eluding oneself”.^30^ Second, as Billon (2014, Sec. 5.1) shows in detail, many Cotard patients are uncomfortable using the word “I”, sometimes preferring “he” or “she” or “it”, proper names or nicknames (“Madam Zero”), or complicated periphrases. This might be taken as a further indication that the underlying “experience of a ‘me’” (Simeon and Abugel 2006: 143–4) is lacking. Again, this is not matched by the linguistic behaviour of patients with thought-insertion.

Both the depersonalisation spectrum, and thought-insertion, leave for-me-ness intact while affecting subjective character in some other way. Accepting my tripartite distinction allows us to say what way that might be in each case. The level of detail required to keep separate what are thought to be different nosographic kinds is thus a further motivation for adopting a threefold approach.

Of course, the psychiatric conditions described in this section are complex and difficult to understand, and a proper defence of the characterisation of depersonalisation and thought-insertion I propose here would require a much more detailed study. The main point of this empirically-informed discussion, however, isn’t so much to argue that the real-life phenomena under consideration *are* indeed best interpreted as I suggest. It is, rather, to show that such an interpretation is *consistent*; and that we can thus conceive of possible cases where for-me-ness, me-ness and mineness fail to be instantiated together, whether or not the real pathologies mentioned above turn out to be just such cases. The three concepts accordingly correspond to different properties, properties which *could* fail to be all satisfied by the same particular. In addition, there is at least some initial appeal to the suggestion that they also fail to be jointly satisfied in some pathological cases in reality.

### For-me-ness, Me-ness and Mineness in Normal Experience

None of this, it should be emphasised, rules out that all the ordinary experiences of normal subjects exhibit conjointly for-me-ness, me-ness and mineness. In fact, I think there is reason to believe this to be the case.

Here is an argument from justification. In ordinary circumstances, when I have an experience – say, a tactile experience of the cat’s silky fur – this as such gives me some justification for making a number of judgments. These include at least: (i) the judgment that the cat has silky fur; (ii) the judgment that an experience to this effect is present; (iii) the judgment that I am present; (iv) the judgment that the experience is mine.

It has been argued^31^ that experiences, furthermore, give *immediate* justification for judgments such as (i). Here, I use the notion of ‘immediate justification’ as defined by Soldati (2012).^32^ To say that the perceptual experience provides the subject with immediate justification to judge (i) is to say that it gives her “a kind of warrant that does not depend on, for instance, any further inferentially acquired justification.” The experience as of the cat’s fur being silky can be used as evidence that the cat’s fur is silky, “without having to rely on any further evidence” to support the experience itself. The experience is a reason to judge the cat’s fur to be silky that is not itself in need of further justification. Note that this notion of immediate justification concerns the type of the epistemic warrant, not the *strength* of this warrant, or the *psychological capacities* involved in arriving at it. Justification that is immediate in this sense does not have to be infallible; in the case of perceptual experience, it is in fact typically defeasible. And to say that the experience *warrants* the corresponding judgment immediately is not to say that the judgment is *arrived at* in a way that is psychologically immediate; it does not, in particular, rule out that conceptual capacities are involved in the process, as they surely are, or that we need to have other beliefs in other to acquire the relevant concepts. The immediacy I have in mind characterises the (normative) *justification* relation between two states (the experience and the judgment), not their (factual) *psychological* relation.

Now, I think that at least as good a case^33^ can be made that experience also immediately justifies judgments (ii), (iii) and (iv). Suppose I am now undergoing some experience or other, e.g. feeling the cat’s smooth fur. The mere presence of this experience, as such, is evidence for judging (ii) that the experience is present, (iii) that I am present, and (iv) that the experience is mine. Having an experience is enough to be licensed to judge it is happening, to self-attribute it, and to judge that I exist. When I reflect on the experience and make those judgments, if asked for my *reason* to do so, it is hard to see what else I could invoke than the experience itself. Again, this does not mean that my *capacity* to make the judgments is unmediated; only that the relevant epistemic *warrant* is provided by the mere having of the experience. It is *something about the experience*, something intrinsic to it, that supports judgments (ii), (iii) and (iv). This I take to be at least a *prima facie* reason to think that we typically have *experiential* access to the experience, to ourselves, and to the fact that the experience is ours; or, in my terminology, that the phenomenal character of a normal experience includes for-me-ness, me-ness, and mineness.

Further support for this claim is offered by a contrast with blindsight patients.^34^ These patients have large blind areas in their visual fields, but are somehow able to respond correctly to visual stimuli located in those areas. No visual phenomenology, however, accompanies those responses. Whether or not it is appropriate to say that blindsight patients *have* visual experiences in those instances, it seems doubtful that they would in any case be *immediately* justified in judging experiences to be present, or in self-attributing those experiences. Their justification would have to proceed through a more circuitous route. Let us imagine a ‘super-blindsight’^35^ patient, trained to regularly form beliefs about the objects in her blind field. She could notice that she has the *belief* that a certain object is in her blind area; use her background understanding of her condition, and her knowledge that when she has this sort of belief, it is usually because a form of visual perception she is unaware of is taking place; and infer on this basis that she must be “experiencing” the object in question. This wouldn’t be an *immediate* justification, directly based on the mere *having* of the experience itself and nothing else, as is the case in normal subjects. But note that the only difference between the ‘super-blindsighter’ and a normal subject is that the latter enjoys visual phenomenology, while the former does not. So when a normally-sighted subject has a visual perception, whatever in the experience gives her immediate justification to judge that an experience is occurring, that she herself is present and that the experience is hers must be exclusively based on the *phenomenal* character of the experience. This I take to be a good reason to think that normal experience displays for-me-ness, me-ness and mineness.^36^

### Taking Stock

What precedes makes it reasonable, I believe, to hold the following. (i) *For-me-ness* is plausibly present wherever there is phenomenal consciousness. It is extremely hard to imagine a case where this wouldn’t be true; and it might be a conceptual truth, our main handle on the notion of phenomenal awareness consisting in spelling it out in terms of “there being something it is like *for me*” to have an experience. (ii) On the other hand, *mineness* (in the case of inserted thoughts) and even *me-ness* (in the case of depersonalised and Cotard patients) might disappear from phenomenal consciousness in some pathological contexts. At the very least, it is conceivable that they *could* disappear, as attested by the fact that the proposed interpretation of the pathological cases is coherent. This supports the thesis that different properties correspond to the three notions. (iii) Nonetheless, all three properties are likely present in normal experiences.

Now the fact that the terms standing for subjective character are used in a way that betrays a lack of discrimination between the three different notions is certainly due to the fact that writers take all three concepts to describe adequately phenomenal awareness as they encounter it, i.e. in ordinary cases. And so it probably is, as I have just argued. But that would be a substantial result, and not a conceptual truth. Every time a move is made from for-me-ness to me-ness or mineness, an argument must be produced. But such an argument will very often be found to be lacking, and an equivalence to be presupposed – wrongly, as I hope to have shown; and to damaging effect for existing arguments, as I will argue in what remains of this essay.

## A Confusion Affecting Argumentative Practice: Kriegel and Zahavi

The aim of my observations is not merely a cautionary one, i.e. to urge for terminological regimentation, lest we fell into equivocations that would invalidate our reasoning on subjective character. It is also critical; the confusion, as I submit in this final section, already has damaging effects on existing arguments.

Three of the four arguments I review are taken from works by Kriegel or Zahavi. This isn’t to suggest that the conflation I hope to have identified is specific to them. The examples below are representative of a much more widespread tendency in the literature. I concentrate on those two writers because they, with a few others, have greatly contributed to advancing our understanding of subjective character in recent years. Rather than singling them out for criticism, the focus reflects the importance of their work on the issue.

A first example of an argument undermined by the conceptual shift was already analysed in Section 3.6.2. An argument offered by Kriegel (2004) in support of the claim that a state being conscious at all depends on peripheral self-consciousness on the part of the subject was found to be question-begging, assuming as it does the equivalence between for-me-ness and mineness.

A second illustration was offered in Section 4.2, in the discussion of schizophrenic thought-insertion. According to the ‘simple account’ of the phenomenon, this is a case where experiences come without a “sense of ownership”. A widely accepted argument against this interpretation invokes the observation that a patient with alien thoughts still has “first-person access” to them. I suggested that the argument trades on an unwarranted equation between for-me-ness and mineness, and has accordingly little weight.

A third example is to be found in Zahavi and Kriegel (2015). The article introduces subjective character in terms befitting *for-me-ness*: “Compare your experiences of perceiving an apple and remembering a banana. […] the two experiences have something very fundamental in common: in both cases it is *for you* that it is like something to have them.” They go on, however, to use this notion indiscriminately with what is really *me-ness*, talking of a “minimum point of self-awareness” involved on the part of the subject by virtue of the fact that her experiences are given to her “in a distinctly first-personal way”. This shift proves damaging for at least one of the authors’ arguments.

The authors consider in Section 4 the ease with which we are able to report on our experiences. You are presently absorbed in reading this text; if suddenly “asked what experience [you] are having, [you] can respond immediately and effortlessly.” In addition, there is “never any sense of surprise regarding what the experience is; instead, there is a sense of familiarity.” Zahavi and Kriegel propose that the best explanation for this ease of reportability, and for the feeling of familiarity, is that my awareness of the experience is part of the experience itself. In other words, the experience includes *for-me-ness* as part of its phenomenal character:

[…] the best explanation of the sense of familiarity with, and lack of surprise regarding, my concurrent experience is that I was aware of it all along, in that it is built into the very phenomenal character of the experience that it is like something *for me*. (Zahavi and Kriegel 2015: 46)

The phenomenal datum that is for-me-ness grounds another phenomenal datum, namely the feeling of familiarity with our experiences, and also serves as the categorical basis of a disposition, namely our capacity to report the experiences with ease.

So far, so good. However, the authors go on to rephrase this very convincing point by saying that for-me-ness is a categorical basis for a *distinct* disposition, namely our capacity for first-person thought:

Our own explanation is that this ever-present sense of familiarity and lack of surprise is grounded in the ubiquitous for-me-ness of experience, which itself is the categorical basis of one’s capacity for first-person thought in the right kind of creatures. (*Ibid*.: 47)

The standard meaning of “first-person thought” identifies it as a thought about the subject entertaining it, typically a self-attribution of the form “I am F”. The suggestion, here, is that for-me-ness grounds the capacity to form thoughts (and reports) in which we self-attribute our experiences, e.g. “*I* am consciously reading”, “*I* am smelling the dog’s fur”, etc. This is quite different from saying that for-me-ness grounds our capacity to report the mere presence of the experience. While I find great intuitive appeal to the weaker, initial claim (that a *phenomenal* awareness of our experiences grounds our capacity to *think* about them), I think that the more substantive one to which the authors transition without warning (that a phenomenal awareness of our *experiences* grounds our capacity to think about *ourselves*) would require much additional support.^37^ As I hope to have shown in Section 3.4, nothing in the notion of for-me-ness, as such, allows us to treat it as an awareness the subject has *of herself*. Even if we accept that for-me-ness is the categorical basis for our capacity to judge a certain experience to be present, it doesn’t follow, without further argument, that this very same property is also the categorical basis of our capacity to judge that *we* are present, and *own* the experience. Distinct phenomenal properties, namely me-ness and mineness, would seem more suited to ground these further dispositions.^38^ It is likely that Zahavi and Kriegel’s failure to distinguish between the three different notions, and corresponding properties, is what leads them to present a stronger claim as a mere reformulation of the weaker one they previously argue for.

A fourth and final example of the effects of the confusion is to be found in an argument from the presence of for-me-ness in phenomenal consciousness, to the conclusion that there exists such a thing as a “core” or “thin” or “minimal” self within the stream of consciousness. This line of thought is familiar from the works of Zahavi (2005, Chapter 5), G. Strawson (1997, 2009), Damasio (1999), Gallagher (2000), and others. The inference is problematic, whether it is taken to yield, by order of increasing strength, an *epistemic* thesis (we have an awareness of the self in having experiences), a *phenomenal* thesis (this access to the self is of the phenomenal kind), or a *metaphysical* thesis (this “self-experience” *is* the self, or at least *a* form of selfhood). Zahavi (2005) commits to all three claims, as the characteristic passage below makes clear:

[…] the self is claimed to possess experiential reality, is taken to be closely linked to the first-person perspective, and is, in fact, identified with the very first-personal *givenness* of the experiential phenomena. [...] [T]he most basic form of selfhood is the one constituted by the very self-manifestation of experience. To be conscious of one*self*, consequently, is not to capture a pure self that exists in separation from the stream of consciousness, but rather entails just being conscious of an experience in its first-personal mode of givenness; it is a question of having first-personal access to one’s own experiential life. Thus, the self referred to is not something standing beyond or opposed to the stream of experiences but is rather a feature or function of its givenness. In short, the self […] is taken to be an integral part of our conscious life with an immediate experiential reality. (Zahavi 2005: 106)

When undergoing an experience, one is “conscious of oneself” (*epistemic thesis*); nothing more is needed for this awareness of oneself than “being conscious of an experience in its first-personal mode of givenness” (*phenomenal thesis*); furthermore, “the self referred to” just *is* (“is identified with”) “the very first-personal givenness of the experiential phenomena” (*metaphysical thesis*).

Now, as I argued in Section 3.4 and 3.5, the “self-manifestation of experience” that Zahavi invokes, i.e. the fact that it manifests itself *to me* (*for-me-ness*), does not entail that I am thereby aware of myself in any way. The uncontroversial observation that experiences are special to their subject, in that they are an object of “first-personal” access to her, cannot be equated with the much more contentious thesis that the experiencing subject is thereby self-aware (*cf*. Section 3.4). Zahavi’s transition from the subjective character of experience to an awareness of the self in experience is thus a great leap, but one that he considers as relatively innocuous precisely because he fails to distinguish between subjective character as *for-me-ness*, as *me-ness*, and as *mineness*. As the assumption of an equivalence is unwarranted, the argument from the “self-manifestation” of experience (*for-me-ness*) to a phenomenal access to the self (*me-ness)* doesn’t go through. As to the metaphysical thesis that the “me-ish” quality of experience *is* the self, it succumbs to the same objection as the phenomenal thesis in so far as it presupposes it. Both theses might still be true (I gave an argument for the phenomenal thesis in arguing for the presence of me-ness in normal experience in Section 4.4); but they cannot be established simply on the basis of our noticing that there is something it is like *for us* to experience.

## Conclusion

In this essay, I argued that much of the debate on the subjective character of experience is affected by a failure to distinguish between three different dimensions one might identify as the invariant phenomenal core across all experiences: that my experiences appear to me (*for-me-ness*); that I am manifested to myself through them (*me-ness*); and that they are presented as my own (*mineness*).

I showed that the three corresponding notions do not form a class of mutually equivalent concepts. For-me-ness, at the very least, entails neither me-ness nor mineness. I also gave some reason to doubt whether the complementary relations of entailment hold. The previous point, in any case, suffices to preclude conceptual equivalence between all three notions.

In addition, I argued that the non-equivalent notions correspond to distinct properties, which *could* fail to be instantiated simultaneously. That this is at least conceivable is showed by presenting an interpretation of some mental pathologies where for-me-ness could be thought to occur without mineness (schizophrenic thought-insertion) and with neither mineness nor me-ness (depersonalisation and Cotard syndrome). Whether or not the interpretation is correct (which would mean that the three properties are not always jointly present in the actual world), it is at least coherent (which means that they are distinct properties).

That different notions as well as different properties are being conflated in discussions of subjective character invites greater caution in the future, and I recommend the use of distinct terms to keep track of the three dimensions. This essay also amounts to a critique of all arguments trading on the illegitimate assumption of an equivalence between for-me-ness, me-ness and mineness. I analyse in detail four examples of arguments guilty of this, and found them accordingly invalid.

## Footnotes

1 See also Zahavi (1999) and (2005: 123) on subjective character. Other contemporary authors making use of related notions include those cited in Section 1.2, as well as Shoemaker (1996: 157); G. Strawson (1997, 2009); Gallagher (2000); Neisser (2006, 2008: 56–8, 2014, 2015); Drummond (2006: 200); Hohwy (2007); Grünbaum (2012); Ganeri (2012: 151 sq.); Williford (2015). This representative list is far from exhaustive.

2 Kriegel (2009: 101); my emphasis.

3 I ignore a complication here: among those who think that the subject’s relevant awareness of a given mental state, *m*, is to be understood as the result of *m* being the object of a mental state *m’*, there is a dispute as to whether *m’* is a *distinct* mental state, or is *identical* to *m*. “Higher-order” theorists (e.g. Rosenthal 1986) defend the first option, while “same-order” theorists (e.g. Kriegel 2006) advocate the second option. To simplify, I talk here as if the same-order approach were right; however, nothing in what follows hangs on this. What matters here is just that the *target* of the relevant awareness is the conscious experience, *m*.

4 Forebears to the view include Sartre (1943: 19–20), Brentano (1874/1973: 153–4), and perhaps Aristotle – see Caston (2002).

5 The expression is Kriegel’s. See his (2009), Chapter 4, in which he gives a perceptive critical account of the view.

6 Cf e.g. *Principles of Philosophy*, Part I, §9, in Descartes (1996) VIIIA p. 7–8.

7 Doing justice to the richness and complexity of the historical roots of the notion of subjective character as me-ness would far exceed the limits of this paper, and would constitute a distinct project. The goal here is simply to highlight an existing use of the notion of subjective character, and to argue that it is conceptually distinct from the other two common uses I identify in sections 2.2 and 2.4.

8 See also Neisser (2006) for a similar distinction.

9 See also e.g. P. F. Strawson (1966: 165).

10 I am grateful to Lucy O'Brien for pointing this out to me.

11 E.g. Dretske (1995), Tye (1995), Martin (2002).

12 But see 3.5 below.

13 More on Williford (2015) in 3.5 below.

14 Dainton (2008, Sections 8.1 and 8.2) and Peacocke (2014) both make a detailed case that we can conceive a creature endowed with conscious states – states that are ‘for’ the creature – but with no phenomenal awareness of itself whatsoever – no me-ness.

15 Kriegel, for instance, appears to restrict the scope of what is covered by the state’s awareness of itself to its *semantic* properties.

16 I am grateful to an anonymous reviewer for inviting me to discuss this issue.

17 The “pearl view” of the self advocated in G. Strawson (1997) bears a resemblance to this type of minimalism, but the close relation it describes between self and experience arguably falls short of *identity*. Minimal notions of selfhood are also to be found in Damasio and Gallagher, among others.

18 Williford (2015: 9/27), quoted in 3.4 above.

19 I am not sure whether it could additionally be argued, on such views, that for-me-ness entails mineness, if only because it is doubtful whether we can still talk of the subject *owning* her experience, if she is *identical* with it (or an aspect of it).

20 As I stressed above, this is not the case of Williford.

21 More on this in 5.2 below.

22 My emphasis.

23 The second argument, reprised from Rosenthal, is that one can’t think at all of a *particular* mental state (as opposed to the *kind* of mental state it is an instance of) without thinking of the subject of that state. I don’t see any reason to accept this. Another way to think of a token mental state in an individuating manner is to think of its time and place of occurrence. Yet another way is to think a singular or “*de re*” thought about it, which we can do by being acquainted with it, whether or not we associate any individuating descriptions with it.

24 Neisser (2015), especially Chapter 3, offers an extended analysis and critique in the same spirit.

25 All the reports are quoted by Billon (2014); in this first quote, the translation from the French is also his. Billon argues that the depersonalisation syndrome is evidence for the possibility of phenomenal consciousness without “subjective character” (2014). I take him to use the term in a way that encompasses at least me-ness and mineness as defined here, although he doesn’t make those further distinctions. His interpretation of the data he reviews might be compatible with my point here, namely that the absence of subjective character in both those senses doesn’t entail the absence of for-me-ness. (But see Billon 2014: 21–4 for qualifications.) Putting the claim in this way has the advantage of not equating it to the claim that phenomenal awareness can occur without subjective character *in any sense of the term*. In my interpretation, *some* kind of subjective character (namely for-me-ness), although not all of its three forms, is retained in the experience of depersonalised and Cotard patients.

26 For a review of the debate, see Bortolotti (2010), Chapter 5.

27 By contrast, as Billon (2014, Section 5.1) stresses, some Cotard patients avoid using “I”, “me” and other first-person expressions as much as possible, preferring proper names, third-person expressions and periphrases. This might be taken as further evidence that, in their case, *me-ness* as well as mineness is lacking. More on this below.

28 See Billon (2011) for a different defence of the simple account.

29 Janet (1928: 43), quoted and translated by Billon (2014).

30 Unless they also suffer from depersonalisation, which can happen.

31 See e.g. Soldati (2012). Pryor (2005) and Goldman (2008), among others, also argue that there is immediate justification.

32 A related notion of immediate justification can be found in Pryor (2005).

33 As for (i), some might argue that further beliefs about the regular, reliable correlation between experiences and what they represent in the world also need to be in place to warrant perceptual judgements, at least as background assumptions. I will remain neutral on this point. In any case, there is no room for a similar worry when we consider (ii), (iii) or (iv), as no scenario of the kind invoked by Cartesian scepticism seems available that would make us doubt the experience as evidence for its own existence, for my existence, or for the fact that the experience is mine.

34 For a detailed presentation of the condition, see e.g. Weiskranz (1986/1998).

35
*Cf*. Block (1995). Blindsight patients can make correct guesses about the objects in their blind field when prompted, but do not spontaneously form beliefs about those objects.

36 I am grateful to Conor McHugh for helping me articulate this argument from justification, and for suggesting the contrast with the blindsight case, which Alexandre Billon helped me sharpen. I am also grateful to an anonymous reviewer for prompting me to spell out the argument more fully. Were the argument to be found unconvincing, please note that this wouldn’t weaken in any way the primary thesis of this essay, i.e. that for-me-ness, me-ness and mineness are not a set of conceptual equivalents (whether or not they normally co-occur).

37 As an anonymous reviewer pointed out, the distinction between those two abilities might be related in some way to the distinction between the two uses of the first person identified by Wittgenstein (1958), namely the use of “I” ‘as subject’ *vs*. its use ‘as object’. Exploring further this connection, or the specific way in which each ability is grounded in the subjective character of experience, would however take me beyond the limited scope of this essay. Links between the “first-person perspective” built into experience, and the capacity to use the first person to self-refer, have been explored by Shoemaker (1996), Evans (1982, Chapter 7), Campbell (1994), Bermúdez (1998), Neisser (2008: 56 sq.), Grünbaum (2012), among many others.

38 This is precisely the theory I defend in Guillot (2012).
